# A Simplified and Effective Approach for the Isolation of Small Pluripotent Stem Cells Derived from Human Peripheral Blood

**DOI:** 10.3390/biomedicines11030787

**Published:** 2023-03-05

**Authors:** Eirini Filidou, Leonidas Kandilogiannakis, Gesthimani Tarapatzi, Michail Spathakis, Colin Su, Alin Rai, David W. Greening, Konstantinos Arvanitidis, Vasilis Paspaliaris, George Kolios

**Affiliations:** 1 Laboratory of Pharmacology, Faculty of Medicine, Democritus University of Thrace, Dragana, 68100 Alexandroupolis, Greece; 2 Tithon Biotech Inc., San Diego, CA 92127, USA; 3 Baker Heart and Diabetes Institute, Melbourne, VIC 3004, Australia; 4 Baker Department of Cardiovascular Research Translation and Implementation, La Trobe University, Melbourne, VIC 3086, Australia; 5 Baker Department of Cardiometabolic Health, University of Melbourne, Melbourne, VIC 3052, Australia; 6 Central Clinical School, Monash University, Melbourne, VIC 3004, Australia

**Keywords:** pluripotent stem cells, PTH1R, small blood stem cells, stem cell isolation, VSELs, Yamanaka factors

## Abstract

Pluripotent stem cells are key players in regenerative medicine. Embryonic pluripotent stem cells, despite their significant advantages, are associated with limitations such as their inadequate availability and the ethical dilemmas in their isolation and clinical use. The discovery of very small embryonic-like (VSEL) stem cells addressed the aforementioned limitations, but their isolation technique remains a challenge due to their small cell size and their efficiency in isolation. Here, we report a simplified and effective approach for the isolation of small pluripotent stem cells derived from human peripheral blood. Our approach results in a high yield of small blood stem cell (SBSC) population, which expresses pluripotent embryonic markers (e.g., Nanog, SSEA-3) and the Yamanaka factors. Further, a fraction of SBSCs also co-express hematopoietic markers (e.g., CD45 and CD90) and/or mesenchymal markers (e.g., CD29, CD105 and PTH1R), suggesting a mixed stem cell population. Finally, quantitative proteomic profiling reveals that SBSCs contain various stem cell markers (CD9, ITGA6, MAPK1, MTHFD1, STAT3, HSPB1, HSPA4), and Transcription reg complex factors (e.g., STAT5B, PDLIM1, ANXA2, ATF6, CAMK1). In conclusion, we present a novel, simplified and effective isolating process that yields an abundant population of small-sized cells with characteristics of pluripotency from human peripheral blood.

## 1. Introduction

Regenerative medicine is an ever-growing scientific field that employs stem cells for the generation of differentiated cells and/or tissues with possible applications in clinical practice [1,2,3]. Stem cells are characterized by three main traits, their self-renewal, their differentiation capacity and their regenerative potential, and can be categorized based on either their differentiation potential or the tissue origin [4,5,6]. Depending on their differentiation potential, stem cells can be categorized as totipotent, pluripotent, multipotent, oligopotent or unipotent [7,8,9]. Regarding tissue origin, the two major categories are embryonic and somatic stem cells. Embryonic stem cells are pluripotent, having significant potential for self-renewal and cell differentiation, whereas somatic stem cells are more confined, and their characteristics vary depending on the tissue origin [10,11,12]. Thus, for many years, there has been a great interest in embryonic stem cells, as they can be excellent candidates for applications in regenerative medicine, such as cell-based therapy [13,14,15]. Nonetheless, there are ethical and experimental limitations to using these cells, including scalable, efficient isolation [16,17].

In adults, stem cells can be found in various tissues, such as the bone marrow, peripheral blood, adipose tissue, intestine and many others, but, in most cases, they are characterized as multipotent, giving rise to limited types of different cells [18,19]. Hematopoietic, mesenchymal and epithelial stem cells are the three major multipotent stem cells found in adult tissues [11,12,20]. Although most multipotent stem cells can be easily isolated and cultured, their use in regenerative medicine is limited due to their restricted differentiation capacity [16,20]. Therefore, regenerative medicine requires stem cells that are pluripotent, can be easily obtained in large numbers and do not pose ethical constraints.

The discovery of induced pluripotent stem cells (iPSCs) was considered a milestone in regenerative medicine because, for the first time, researchers had the opportunity to utilize a natural source of adult pluripotent stem cells [21,22]. iPSCs were generated in vitro by introducing four transcription factors, OCT4, SOX2, MYC and KLF4, the “Yamanaka factors”, into differentiated adult cells, thus reversing the cell fate and resulting in pluripotent undifferentiated stem cells [21,23,24]. One of the main advantages of iPSCs was the possibility of having an unlimited supply of autologous cells, with several clinical applications, either as undifferentiated stem cells or differentiated into other adult cell types and, most importantly, without the fear of immune rejection [25,26]. Nonetheless, iPSCs also presented several challenges, with the most significant ones being their high cost and manufacturing time, and thus, in most cases, iPSCs were clinically impractical to use [27,28]. One possible solution was the generation and banking of donor iPSC cell lines, which can give rise to allogeneic tissue-matching cell products. Although this approach resulted in a significant reduction in the manufacturing time, it is still a relatively high-cost procedure, which also demands the use of immunosuppressive agents when clinically applied [29,30,31]. Therefore, the ultimate solution to the aforementioned challenges would be the discovery and identification of a naturally occurring and autologous pluripotent stem cell population, which could be found in abundance and have low cost and processing time [32,33].

Fifteen years ago, the group of Ratajczak identified in adults a population of very small cells bearing markers of pluripotency, such as stage-specific antigen (SSEA), nuclear Oct-4A, Nanog, and Rex1, and thus, named these cells “very small embryonic-like” (VSEL) stem cells [34,35]. VSELs have a size of 5–7 μm in humans, are thought to give rise to various cell types (including hematopoietic, mesenchymal and endothelial stem cells) and are believed to act as a backup regeneration for adult tissues [36,37]. Nonetheless, isolation of VSELs from peripheral blood has since remained a challenge due to their small cell size and the limited number of recoverable cells [38,39]. Most of the isolation techniques use density gradients or reagents for erythrocyte lysis [40,41,42] that not only results in a limited number of isolated cells but also makes their isolation complex and time-consuming. Therefore, to take advantage of the regenerative and translational potential of VSELs, we require a streamlined, efficient approach to gain this pluripotent blood-derived stem cell population.

Taking into consideration all the aforementioned complications in isolating VSELs, this study employed a simplified approach using processing steps of centrifugation and filtration of fresh whole blood, without the use of density gradients and immuno-cell separation methods, in order to isolate and biochemically characterize small blood stem cells (SBSCs) from peripheral blood.

## 2. Materials and Methods

### 2.1. Healthy Volunteers

Peripheral blood was collected from healthy volunteers, who were informed and agreed to participate in this study by giving their written consent. All principles outlined in the Declaration of Helsinki for all human experimental investigations were followed. Healthy volunteers were of all sexes and their ages varied from 20 to 30 years old.

### 2.2. Isolation of SBSCs

Isolation of SBSCs was performed with the following steps. Briefly, 30 mL peripheral blood was collected in tubes containing sodium citrate and the tubes were then centrifuged at 600× *g* for 10 min to separate red blood cells from the plasma layer. The plasma layer was then carefully transferred into a new centrifuge tube and centrifuged again at 1200× *g* for another 10 min. Supernatant plasma was then discarded, and the pellet containing the SBSCs was resuspended in 1 ml of sterile saline. Finally, the suspension was filtered through a 5 µm syringe-driven filter and SBSCs were collected for further studies.

### 2.3. SBSCs Counting

SBSCs were isolated, as described above, from healthy young volunteers, and appropriate dilutions with sterile saline were made. SBSCs were then stained with 0.4% trypan blue solution (Gibco™—Thermo Fisher Scientific, Waltham, MA, USA) in order to exclude any possible dead cells and debris, and a 10 µL of the sample was loaded on each loading groove of the Neubauer chamber. Using a 40× magnification, the five RBC squares of the counting chambers were identified, and the live population of SBSCs was counted. The final number of live SBSCs was calculated using the formula:

$$\mathrm{SBSCs}\;\mathrm{per}\;\mu L=\frac{\mathrm{counted}\;\mathrm{SBSCs}}{\mathrm{counted}\;\mathrm{surface}\;\times \;\mathrm{chamber}\;\mathrm{depth}\;\times \;\mathrm{dilution}\;\mathrm{factor}}$$

Finally, the calculated number of isolated SBSCs was presented as the number of SBCSs per ml of plasma from peripheral blood.

### 2.4. Kyoto Probe 1 Staining

SBSCs were stained using Kyoto Probe 1 (KP-1; Goryo Chemical, Sapporo Japan) according to the manufacturer’s instructions. Briefly, SBSCs were centrifuged at 1200× *g* for 10 min, and the pellet was then reconstituted in phosphate-buffered saline (PBS; Sigma-Aldrich, St. Louis, MO, USA). Next, 10 μg of KP-1 were dissolved in Dimethyl Sulfoxide (DMSO; Sigma-Aldrich, St. Louis, MO, USA) to prepare a 5 mM stock solution and then diluted again in PBS to prepare a 2 μM cell stain solution. The stain solution was then added to the cells, which were incubated for 3 h, and then the culture slides were studied using a fluorescent microscope (Leica DM2000, Leica Microsystems GmbH, Wetzlar, Germany).

### 2.5. Immunofluorescence

SBSCs were characterized using the immunofluorescence method, as previously described [43,44]. Cells were centrifuged at 1200× *g* for 10 min, and the pellet was then reconstituted and fixed in ice-cold 4% paraformaldehyde (PFA; Sigma-Aldrich, St. Louis, MO, USA) for 20 min at 4 °C. After fixation, cells were washed with PBS (Sigma-Aldrich, St. Louis, MO, USA), and then permeabilization was achieved using 0.05% Triton X-100 (Sigma-Aldrich, St. Louis, MO, USA) in 1X PBS for 10 min. To eliminate nonspecific staining, samples were incubated for 60 min with 5% bovine serum albumin (BSA; Sigma-Aldrich, Darmstadt, Germany). Next, antibodies against isotype controls (Supplementary Figure S1) or targets of interest (Table 1) (Supplementary Figure S2) were added at a 1:50 dilution and incubated for 1 h at room temperature. Finally, DAPI (Sigma-Aldrich, Darmstadt, Germany) was added to stain the nuclei, and the samples were observed under a fluorescent microscope (Leica DM2000, Leica Microsystems GmbH, Wetzlar, Germany).

### 2.6. Hematoxylin and Eosin (H&E) Staining

Once isolated, SBSCs were fixed using ice-cold 4% PFA (Sigma-Aldrich, St. Louis, MO, USA) for 20 min at 4 °C. After fixation, cells were washed with PBS (Sigma-Aldrich, St. Louis, MO, USA), and then Hematoxylin (Sigma-Aldrich, St. Louis, MO, USA) was added and incubated for 3 min. Cells were washed with deionized water and then with tap water, and then destain was accomplished using acid ethanol. Cells were washed again with deionized water and then with tap water, and then Eosin (Sigma-Aldrich, St. Louis, MO, USA) was added and incubated for 30 s. Lastly, cells were incubated in ethanol, and the culture slides were studied using a light microscope (Leica DM2000, Leica Microsystems GmbH, Wetzlar, Germany).

### 2.7. Culture of Human Embryonic Stem Cell Line H1

The human embryonic stem cell line H1 (Wicell International Stem Cell Bank, Wicell Research Institute, Madison, WI, USA) was cultured as previously described [45] and served as a positive control in antibody verification of embryonic stem cell markers. H1 stem cells were cultured in 6-well plates coated with Matrigel (BD Biosciences, San Jose, CA, USA), fed daily with mTeSR1 (Stem Cell Technologies, Vancouver, BC, Canada) and observed under a microscope for any spontaneous differentiation. When confluence reached around 90%, H1 stem cells were passaged, seeded in Matrigel-coated chamber slides (ibidi GmbH, Martinsried, Planegg, Germany) and later stained for the detection of embryonic stem cell markers using immunofluorescence.

### 2.8. Isolation of White Blood Cells (WBCs)

Peripheral blood was collected in tubes containing sodium citrate, and equal parts of whole blood and sterile saline were mixed and then carefully applied over Biocoll separating solution (Sigma-Aldrich, St. Louis, MO, USA). Samples were then centrifuged at 1200× *g* for 20 min, and an enriched layer of WBCs was later formed and retrieved using a Pasteur’s pipette. WBCs were then washed twice using sterile saline and used for further studies.

### 2.9. Sample Preparation for Proteomic Analysis

SBSCs were first cultured for 3 days in matrigel-coated (Corning, New York, NY, USA) plates and in DMEM (Gibco Thermo Fisher Scientific, Waltham, MA, USA) in order to discard any inhibitory components found in the blood. In addition, plasma cells, which were obtained after blood centrifugation at 600× *g* for 10 min and removal of red and white cells, served as controls in the proteomic analysis. Plasma cells and SBSCs (n = 4) were then lysed in 1% (*v*/*v*) sodium dodecyl sulfate (SDS; Sigma-Aldrich, St. Louis, MO, USA) and 50 mM HEPES pH 8.0 (Sigma-Aldrich, St. Louis, MO, USA). Sample homogenization was performed using tip probe sonication (Misonix—S-4000 Ultrasonic Liquid Processor). Lysates were quantified with microBCA (Thermo Fisher Scientific, Waltham, MA, USA) and then normalized samples (10 μg protein) were prepared for proteomic analysis as described [46,47] using the single-pot solid-phase-enhanced sample preparation (SP3) method [48]. The samples were reduced (10 mM dithiothreitol, DTT; Sigma-Aldrich, St. Louis, MO, USA) for 45 min at 25 °C and alkylated (20 mM iodoacetamide; Sigma-Aldrich, St. Louis, MO, USA) for 30 min at 25 °C in the dark before the Sera-Mag-based workflow as described before [46,47].

### 2.10. Proteomic Liquid Chromatography-Tandem Mass Spectrometry

Tryptic peptides for one-shot analysis were analyzed on a Dionex UltiMate NCS-3500RSLC nanoUHPLC coupled to a Q-Exactive HF-X hybrid quadrupole-Orbitrap mass spectrometer equipped with nanospray ion source in data-dependent acquisition analysis and positive mode as previously described [46,47,49]. Peptides were loaded (Acclaim PepMap100 C18 3 μm beads with 100 Å pore-size, Thermo Fisher Scientific, Waltham, MA, USA) and separated (1.9 µm particle size C18, 0.075 × 250 mm, Nikkyo Technos Co. Ltd., Tokyo, Japan) with a gradient of 2–28% acetonitrile (Sigma-Aldrich, St. Louis, MO, USA) containing 0.1% formic acid (Sigma-Aldrich, St. Louis, MO, USA), and 28–80% over 56 min at 300 nL min^−1^ at 55 °C (butterfly portfolio heater, Phoenix S&T, Chester, PA, USA). All MS acquisition parameters were as described before [46,47]. Data were acquired using Xcalibur (v4.0, Thermo Fisher Scientific, Waltham, MA, USA). RAW and parameter/processed proteomics data were deposited to the ProteomeXchange Consortium using the PRIDE partner repository and are available in ProteomeXchange with the identifier (PXD039464).

### 2.11. Database Search and Analysis

RAW MS data were processed using MaxQuant [50] (v1.6.14.0) with its built-in search engine Andromeda [47]. Tandem mass spectra were searched as a single batch against the Homo Sapien database (UniProt, UP000005640, Jun 2022; 79,684) supplemented with common contaminants. Specific search parameters included: carbamidomethylated cysteine (fixed modification), oxidation of methionine and N-terminal protein acetylation (variable modifications), trypsin/P (C-terminal to arginine/lysine) with ≤2 missed cleavage sites, FDR at PSM/protein at 1%, initial precursor mass deviation (7 ppm), fragment mass deviation (20 ppm), up to two missed cleavages, with all other parameters at default. Match between runs (MBRs) (time window 1 min) and label-free protein quantitation (LFQ) were enabled. False discovery rate (FDR) at the PSM, protein and site levels were each 1%. Peptides were identified with an initial precursor mass deviation of up to 7 ppm and a fragment mass deviation of 20 ppm. A maximum of two missed cleavages were allowed. The 0.01 Protein groups were analyzed using Perseus (v1.6.7), with contaminants and reverse peptides removed. Comparative analysis based on stem cell marker proteins [51] and abundance treemap using proteomap [52] was performed. Perseus was used to analyze the proteomic data and generate plots [53]. G:Profiler, Reactome and STRING were used for Gene Ontology/functional enrichment analysis.

## 3. Results

### 3.1. Isolation of a Rich and Pluripotent Population of SBSCs Using a Simplified Approach

**Figure 1:**
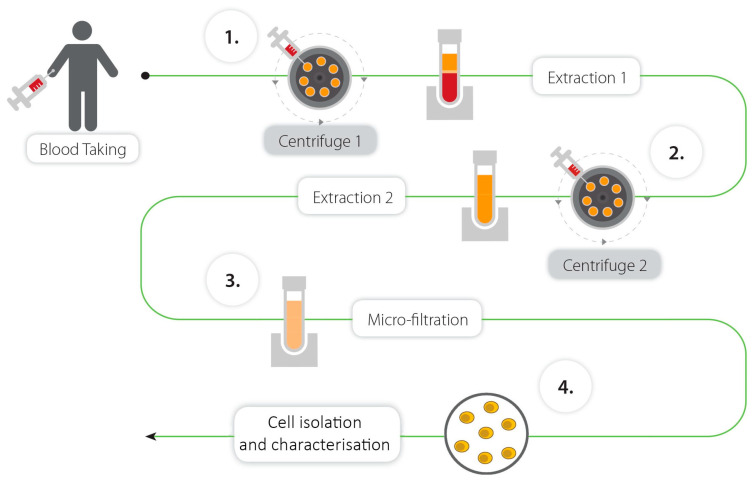
Methodology for the isolation of SBSCs.

SBSCs were isolated from peripheral blood using simple processing steps of centrifugation and filtration that are depicted in Figure 1.

**Figure 2:**
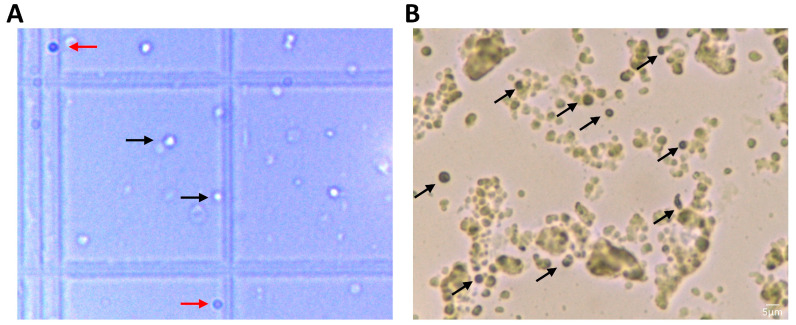
Representative brightfield microscopy image of Trypan Blue-stained and H&E-stained SBSCs. (**A**) Black arrows indicate live cells, while red arrows show dead cells. (**B**) Black arrows indicate H&E-stained SBSCs.

From 30 mL whole blood, our approach resulted in a high concentration of very small cells (about 5 μm), and after staining with trypan blue, over 90% of them were viable (n = 3) (Figure 2A). The mean of the counted live cell population was 2,965,333 cells/mL (SEM: 276,746) of whole blood, and Figure 2B shows a representative field of isolated small stem cells stained with H&E. Black arrows indicate several small stem cells, suggesting that our method can indeed result in high yield isolation.

**Figure 3:**
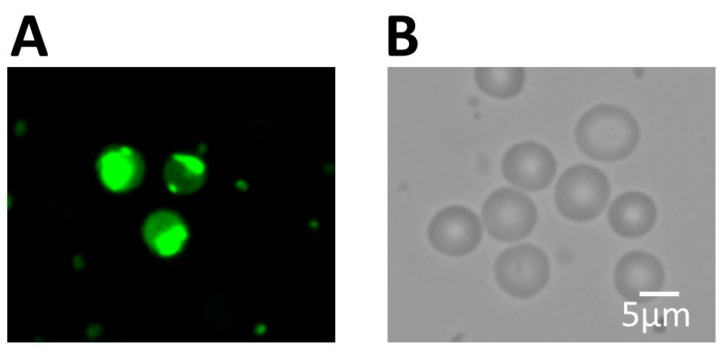
Immunofluorescence and light microscopy images of SBSCs. (**A**) SBSCs stained with KP-1. (**B**) SBSCs observed using a bright field light microscope. Representative 400× snapshots are shown.

Furthermore, we investigated the pluripotency of these isolated cells using staining with KP-1. KP-1 staining detects pluripotent cells, as they fluoresce in the green spectrum due to their lack of ABC transporters, a set of proteins that are normally expressed in differentiated cells, eliminating this dye [54]. We observed positive KP-1 staining of the isolated peripheral blood small cells, suggesting that this population contains pluripotent stem cells (Figure 3A).

To further confirm the pluripotency of our small cell population, we next stained them with antibodies against the pluripotent markers Nanog, CXCR4, SSEA-3 and SSEA-4 and the Yamanaka pluripotent factors Oct4, SOX-2, cMyc and KLF4, known to be expressed in embryonic stem cells [55,56,57,58]. The expression of CXCR4, a marker found not only in embryonic stem cells but also in adult differentiated cells, such as intestinal epithelial cells [59], was also examined. Before proceeding with staining the SBSCs, we tested our antibodies on the H1 embryonic stem cell line and found that they were stained positive for all the pluripotent markers (Supplementary Figure S3).

**Figure 4:**
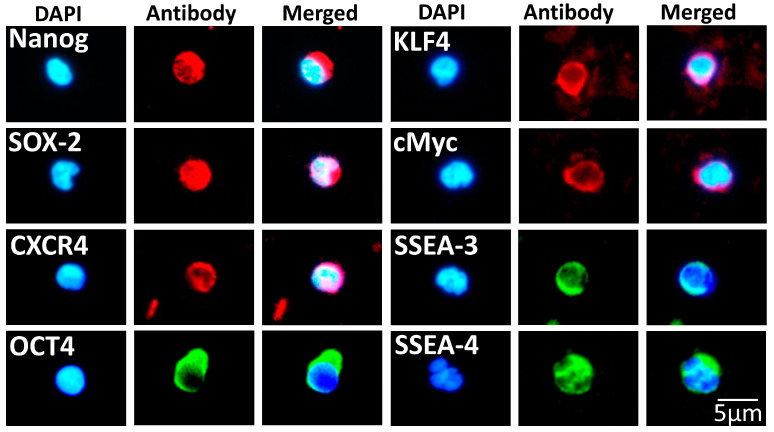
SBSCs stained for embryonic stem cell markers. Representative 400× immunofluorescence snapshots are shown. Isotype control staining was examined for each tested antibody, and the result was negative (Supplementary Figure S1). Blue and red/green colors correspond to DAPI and embryonic stem cell marker antibody staining, respectively.

We observed that some of the isolated cells expressed all the studied pluripotent markers (Figure 4), and although we did not perform any quantitative analysis, the expression of SSEA-3, SSEA-4, Nanog and Oct4 were semi-quantitatively determined to be around 10–20%, 10–20%, 5–10% and 20–30%, respectively, based on the acquired immunofluorescence images. This finding suggests that our novel methodology approach results in isolating a rich population of SBSCs in which some are of pluripotent origin.

### 3.2. SBSCs Express Markers of Mesenchymal Origin

**Figure 5:**
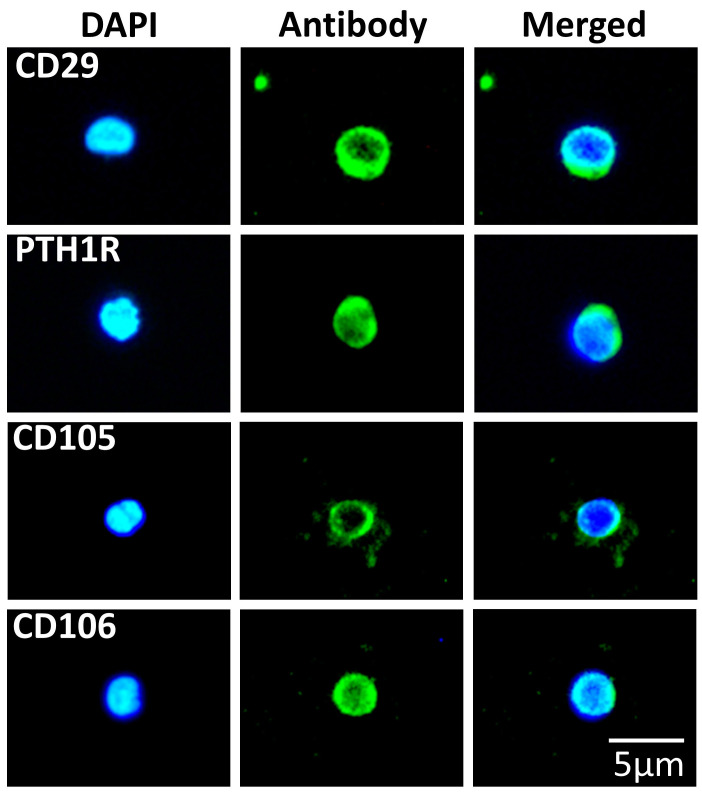
SBSCs stained for mesenchymal stem cell markers. Representative 400× immunofluorescence snapshots are shown. Isotype control staining was examined for each tested antibody, and the result was negative (Supplementary Figure S1). Blue and green colors correspond to DAPI and mesenchymal stem cell marker antibody staining, respectively.

We investigated the mesenchymal characteristics of SBSCs by staining against the known mesenchymal markers, CD29, parathyroid hormone 1 receptor (PTH1R), CD105 and CD106 [60,61,62]. We observed that a portion of SBSCs expressed these markers (Figure 5), and approximately 80%, 90%, 10% and 10% were found positive for CD29, PTH1R, CD105 and CD106, respectively, suggesting that a number of these stem cells may have been differentiated towards mesenchyme lineages.

### 3.3. SBSCs Express Certain Hematopoietic Markers

**Figure 6:**
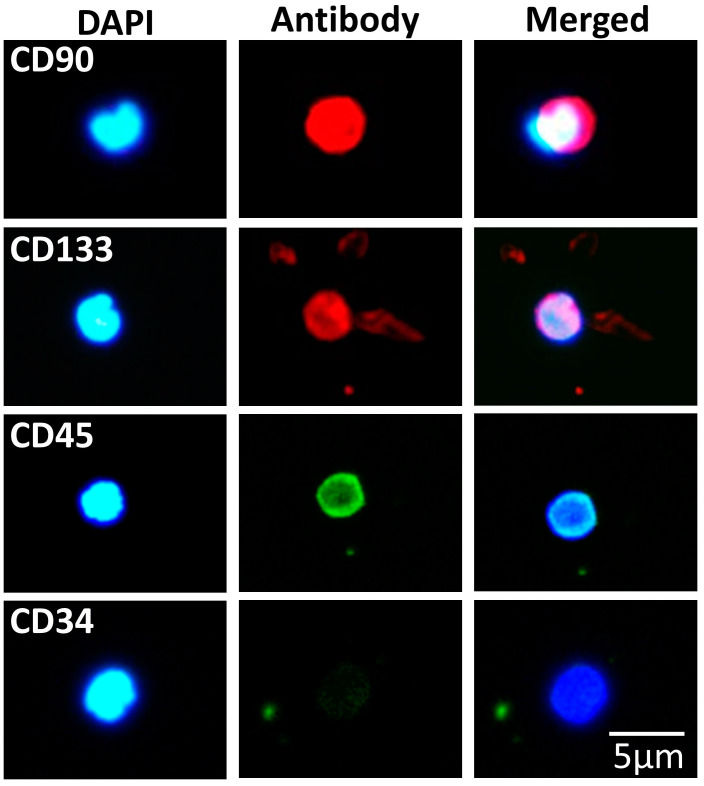
SBSCs stained for hematopoietic stem cell markers. Representative 400× immunofluorescence snapshots are shown. Isotype control staining was examined for each tested antibody, and the result was negative (Supplementary Figure S1). Blue and red/green colors correspond to DAPI and hematopoietic stem cell marker antibody staining, respectively.

We further examined the identity of SBSCs and, in particular, we investigated the possible expression of factors of hematopoietic origin, such as CD90, CD133, CD45 and CD34 [63]. Upon testing, SBSCs were found positive to CD90, CD133 and CD45, but not to CD34 (Figure 6), suggesting that many of the isolated SBSCs were committed towards the hematopoietic lineages.

To exclude any false staining from CD45, we also investigated its expression on WBCs and found that both WBCs and SBSCs were stained positive (Supplementary Figure S4).

**Figure 7:**
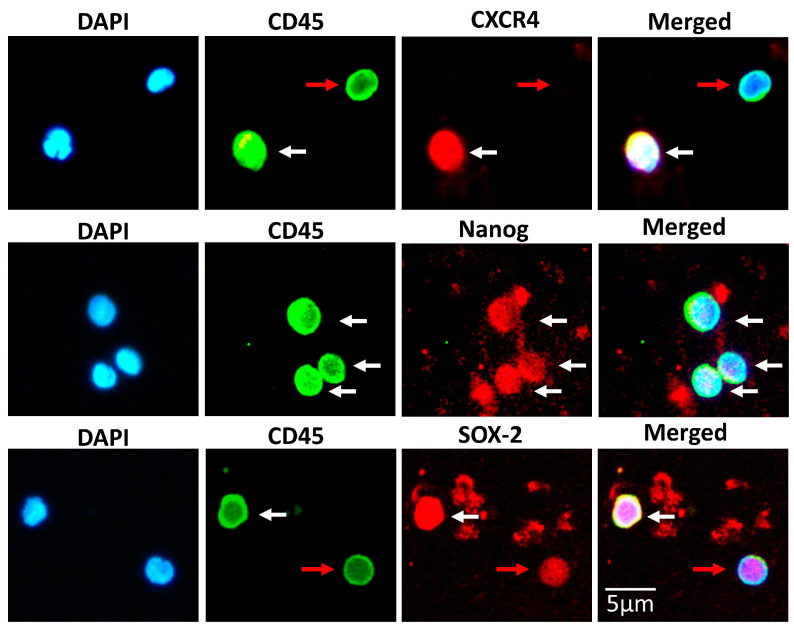
SBSCs double stained for CD45 and embryonic stem cell markers. Representative 400× immunofluorescence snapshots are shown. White arrows indicate simultaneous co-staining of both CD45 and embryonic stem cell markers. Red arrows indicate strong CD45 staining and weak or not at all staining of embryonic stem cell markers. Isotype control staining was examined for each tested antibody, and the result was negative (Supplementary Figure S1). Blue and green/red colors correspond to DAPI and CD45/embryonic stem cell marker antibody staining, respectively.

We further performed a simultaneous double-stain to SBSCs with antibodies against CD45 and the embryonic stem cell markers, Nanog and SOX-2, and the marker CXCR4, in order to investigate whether both pluripotent and already differentiated stem cells co-exist in this isolated stem cell population. Indeed, we observed a mixed population of SBSCs, as most of them expressed both CD45 and the embryonic stem cell markers Nanog, SOX-2 or CXCR4 (Figure 7, white arrows), while a small portion of them expressed CD45 and expressed weakly or not at all the other markers (Figure 7, red arrows). These results indicate that SBSCs are indeed a mixed population of stem cells that may bear both embryonic and hematopoietic markers.

### 3.4. SBSC Proteomics

**Figure 8:**
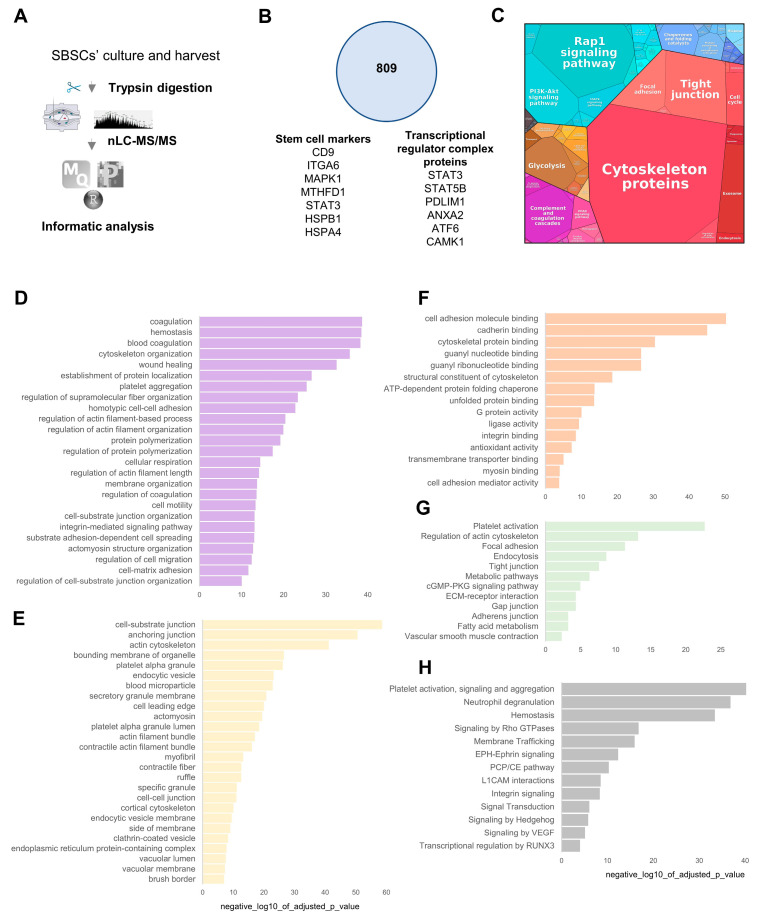
Proteomic analysis of SBSCs. (**A**) Experimental workflow of isolation and proteome analysis of SBSCs. (**B**) Proteome composition of SBSCs, including various stem cell marker proteins, and factors associated with transcriptional regulator complex. (**C**) SBSC proteome abundance treemap, highlighting various adhesion, cytoskeletal, signaling and metabolic factors abundant in SBSCs. The cell size of the SBSC treemap indicates the protein abundance of 809 proteins. (**D**–**H**) Profiler enrichment of the SBSC proteome, Gene Ontology (**D**), Component (**E**), Process (**F**), Function (**G**), KEGG (**H**), Reactome functional enrichments (FDR < 0.05).

To further investigate the expression profile of SBSCs, we performed proteomics analysis of SBSCs and plasma cells (as a control). SBSCs were cultured in DMEM and a pure cell population, free of debris or blood components, was harvested and analyzed (Figure 8A). Proteomic analysis revealed that SBSCs comprised 809 proteins based on stringent inclusion criteria (quantified LFQ, two or more peptides) (Figure 8B, Supplementary Table S1). Various stem cell marker proteins were identified, including CD9, HSPA4, HSPB1, ITGA6, MAPK1, MTHFD1 and STAT3, that were previously characterized either as stem cell markers or as being significant factors for stem cell maintenance [51]. In addition, we report various transcriptional regulator complex proteins in SBSCs, including STAT3, STAT5B, PDLIM1, ANXA2, ATF6 and CAMK1 (Supplementary Table S1). There was strong enrichment for cytoskeletal and adhesion-related components (tight junctions, focal adhesion), and various signaling pathways including Rap1, MAPK and PI3K-Akt in the SBSC proteome (Figure 8C). To gain further insight into the SBSC proteome, functional enrichment analyses were performed, highlighting enrichment in the SBSC proteome indicating source (blood cell, coagulation, platelet), localization (cell-substrate, actin cytoskeleton, membrane), function (adhesion, binding) and pathways associated with metabolism, adhesion, ECM-receptor interaction, platelet activation and membrane trafficking (Figure 8D–H, Supplementary Table S2).

**Figure 9:**
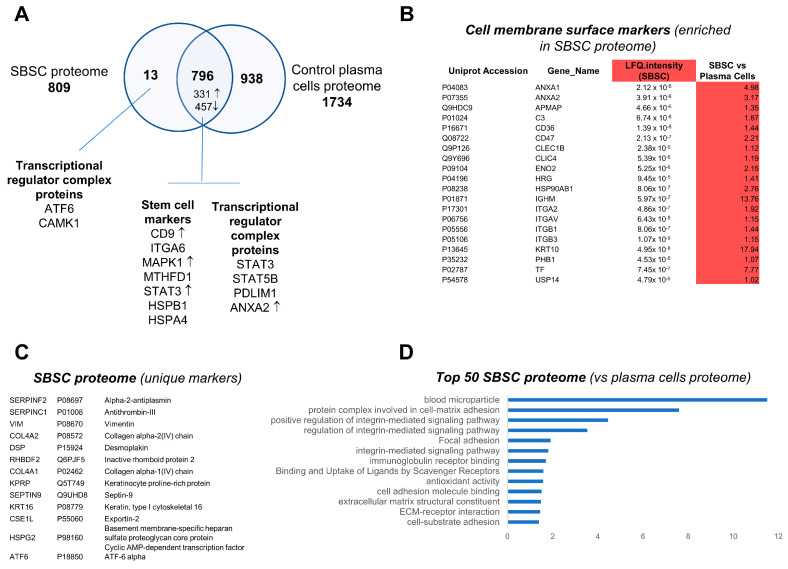
Analysis and comparison of the SBSC and plasma cell proteome. (**A**) Comparison of SBSC proteome with isolated plasma cells (control plasma cells). Proteins uniquely identified in SBSCs (13) and co-identified (796) are shown. Proteins differentially expressed based on LFQ intensity are shown—upregulated expression (↑, LFQ) and downregulated expression (↓) of SBSCs vs plasma cells (average LFQ). Specific proteins identified in each group are indicated, including various stem cell marker proteins, and factors associated with transcriptional regulator complex. (**B**) Enriched cell surface marker proteins in SBSCs (based on UniProt). (**C**) Proteins uniquely identified in the SBSC (vs control plasma cells) proteome. (**D**) Profiler enrichment of top 50 proteins identified in the SBSC vs control plasma cell proteome (LFQ intensity) (FDR < 0.05).

We further compared the SBSC proteome with isolated plasma cells. We show that the SBSC proteome is distinct in the composition of factors associated with transcriptional regulator complex, and differential proteome analysis (based on LFQ intensity) revealed enrichment in various stem cell markers including CD9, MAPK1 and STAT3 (Figure 9A, Supplementary Table S3). Importantly, various cell membrane surface markers were identified enriched in the SBSC proteome (Figure 9B), including various annexins, integrins and Leukocyte surface antigens. Further analysis of proteins uniquely identified in SBSCs revealed their association with transcription factors (ATF6), the extracellular matrix (COL4A1, COL4A2) and membrane/signaling (HSPG2) (Figure 9C). In addition, functional enrichment analyses of the most abundant proteins in SBSCs (LFQ intensity rank, vs Plasma cell proteome, average LFQ) were performed to highlight enrichment in blood microparticle, integrin signaling, ECM/cell-substrate adhesion and antioxidant activity (Figure 9D).

## 4. Discussion

In this study, we report a simplified and efficient approach for the isolation of small stem cells (the SBSCs) from human peripheral blood using straightforward steps of centrifugation and filtration of blood, without the use of density gradients and immuno-cell separation methods as previously reported [64,65]. Our approach results in the isolation of a rich population of SBSCs, with various characteristics, as some of them expressed all the widely studied pluripotent stem cell markers (KP-1, Nanog, Oct4, SOX-2, cMyc, KLF4, CXCR4, SSEA-3 and SSEA-4), other certain hematopoietic markers (CD90, CD133 and CD45) and portions of them being mesenchymal markers (CD29, PTH1R, CD105 and CD106). Therefore, we show that the isolated SBSCs consist of a mixed pluripotent population of stem cells that includes a fraction with mesenchymal and hematopoietic characteristics, aligning with previous studies supporting the heterogeneous character of small stem cells in the cord blood [66,67,68].

More specifically, regarding the embryonic stem cell markers, the KP-1, even though not precisely sensitive for pluripotent stem cell detection, has been reported to be a particularly useful tool for screening undifferentiated pluripotent stem cells [54,69,70]. KP-1 has also been used as the selective part for the creation of synthetic hybrid molecules that can target, attach to, and remove unwanted pluripotent stem cells from cell mixtures, to help avoid tumorigenesis [71]. Our approach resulted in the isolation of a pluripotent subpopulation of SBCSs, positively stained with this specific pluripotent marker, KP-1. The other embryonic stem cell markers that were investigated in this study are widely used in stem cell characterization of pluripotency [55,56,57]. In particular, SSEA-3 was proven to be the selective marker to distinguish the Multilineage-differentiating stress-enduring (MUSE) cells, a pluripotent subpopulation that lies among mesenchymal stem cells and fibroblasts [72,73]. SSEA-4 was one of the embryonic markers [74,75] that characterize primate embryonic stem cells and was used in the characterization and establishment of the well-known embryonic stem cell lines derived from human blastocysts (H1, H9) [76]. Nanog has been described as the new “master gene” of embryonic stem cell pluripotency [77,78,79] and was shown to work together with other key pluripotent factors, such as Oct4 [80,81] and SOX-2 [82,83], to control a set of target genes that have important functions in embryonic stem cell pluripotency [84,85,86,87]. CXCR4, the stromal cell-derived factor 1 (SDF-1) chemokine receptor, is expressed in leukocytes, epithelial cells [59,88] and also in embryonic stem cells [89,90]. These findings strongly suggest the pluripotent character of the isolated SBSCs, as they expressed all the aforementioned markers.

In addition, Oct4, KLF4, SOX-2 and c-Myc have been established as the “Yamanaka factors”, as Yamanaka et al. proved that they are crucial key players in the process of creating induced pluripotent stem cells (iPSCs) [21,23,24,91]. According to the suggestions laid out by the International Stem Cell Banking Initiative, there are specific criteria that should be met before banking an iPSC line [92]. Common characterization methods for pluripotency assessment in establishing iPSC lines include the detection of Nanog and Oct4, as well as the TRA-1-60 and TRA-1-81 cell surface antigens, that are expressed along with SSEA-3, SSEA-4 in human embryonic stem cells and embryonal carcinoma cells [24,93]. A portion of our SBSCs was found to be positive for these Yamanaka markers, and the iPSC pluripotency assessment markers indicated that a subpopulation of them has a strong pluripotent identity.

Apart from the aforementioned embryonic stem cell markers, our proteomic analysis revealed that SBCSs express various proteins (CD9, HSPA4, HSPB1, ITGA6, MAPK1, MTHFD1 and STAT3), which have been previously reported either as stem cell markers or as being significant players in stem cell maintenance [51]. CD9 has been found to be expressed in human embryonic stem cells [94,95] and in mesenchymal stem cells, where its expression was correlated with their ability to proliferate and expand [96], suggesting that its expression in SBSCs may be of equal importance for their proliferation and expansion. HSPA4, a heat-shock protein found to be also expressed in embryonic stem cells [97,98], has been shown to have a critical role in spermatogenesis, as its lack of expression led to reduced numbers of spermatozoa that bore apoptotic characteristics [99], suggesting again that the expression of this marker in SBSCs may be significant for their survival. The expression intensity of HSPB1, another heat-shock protein, has been correlated with the differentiation status of stem cells; HSPB1 is overexpressed in undifferentiating stem cells and downregulated in stem cells already committed towards a differentiation pathway [100,101,102]. ITGA6, an integrin α-6 or otherwise known as CD49f, has been shown to be expressed in various stem cells, having a significant role in self-renewal and proliferation [103,104,105]. MAPK1, otherwise known as Erk2, along with Erk1, are two kinases with significant roles in cell proliferation [106,107,108], and their expression has been shown to be crucial for the survival and self-renewal of hematopoietic stem cells [109,110,111]. Regarding embryonic stem cells, various studies have shown that inhibition of the Erk1/2 signaling pathway is essential for their self-renewal [112,113,114]. MTHFD1 is an enzyme involved in folate metabolism [115,116,117], and although it was reported as a potential stem cell marker by Maguire et al. and Bhattacharya et al. [51,118], there is no other knowledge in regard to its role in stem cell pluripotency, while its polymorphisms have been associated with therapeutic response to methotrexate in childhood acute lymphoblastic leukemia [119,120,121]. STAT3 is well known as a transcription factor for its role in the regulation of the self-renewal of embryonic stem cells [122,123,124], suggesting that its expression in SBSCs may indeed promote their self-renewal.

Identified factors in SBSCs further included proteins associated with cell differentiation. These included LIMS1, HSP90AB1, EIF5A, LYN, S100A4, PA2G4, FYN, CAMK1 and PTBP1, and most of them have an already known role in stem cell differentiation, as seen in Table 2.

Our proteome data also supports the identification (and enrichment to control plasma-derived cells) of various factors highly enriched in SBSCs associated with cell adhesion, matrix localization, antioxidant function and cytoskeleton localization, suggesting that SBSCs might have the potential to migrate through the bloodstream and attach to specific tissues and differentiate towards various cell types. In addition, various signaling pathways including Rap1, MAPK and PI3K-Akt were identified as enriched in SBSCs (Figure 8), as well as integrin signaling factors/pathways highly abundant in SBSCs in comparison to isolated blood cells (Figure 9). These signaling pathways have been previously reported to be also highly enriched in mesenchymal stem cells [168], and more specifically, the MAPK signaling pathway has been found to promote neuronal stem cell differentiation [169]. Rap1 has been shown to regulate the fate of calvarial mesenchymal cells, as it promotes chondrogenesis, while its depletion results in osteogenesis [170]. The PI3K-Akt signaling pathway has been reported to be involved in the differentiation process of mesenchymal cells toward the osteogenic lineage [171].

Regarding the widely studied markers for mesenchymal stem cells, CD29 and CD105 [172,173,174], portions of SBSCs were found to be positive for both, indicating that they may be prone toward mesenchymal lineage differentiation. On the other hand, CD106, which was also found to be expressed in a small portion of SBSCs, is a marker primarily expressed in endothelial cells [175] and secondarily in some cases of mesenchymal stem cells [62,173,176], suggesting that a subpopulation of SBSCs may be committed either towards endothelial cells or specific types of mesenchymal stem cells.

Furthermore, a subpopulation of SBSCs was also found to stain for membrane-bound PTH1R. It was previously proposed that VSELs may have an important role in bone metabolism and hence, it is plausible to suggest that they may express parathyroid hormone (PTH) receptors [177]. PTH receptors have been shown to be expressed in mesenchymal stem cells [178,179,180] and are thought to be important in driving the anabolic effect on the bone that has been observed with intermittent PTH and parathyroid hormone-related protein (PTHrP) agonists [177,181,182]. The fact that these cells seem to be of more primitive origin than mesenchymal stem cells, due to their size, large quantity, and pluripotent nature, suggests that SBSCs may be of paramount importance in bone metabolism.

Finally, the hematopoietic marker CD90, a surface marker of different types of cells, including T cells and mesenchymal stem cells [183,184,185], was expressed in SBSCs, suggesting again that these cells may have a mixed phenotype. Regarding CD133, although it was initially described as a stem cell marker of hematopoietic origin [186,187,188], it was later used for the identification of VSELs [67,189,190]. Our results agree with the aforementioned report on VSEL characterization, as SBSCs were also found to be CD133 positive. In contrast to previous studies on VSEL characterization using CD45 and CD34 markers [34,189], in our study, SBSCs were found to be CD45 positive, but CD34 negative, suggesting that our method may result in the isolation of a different stem cell population. Similar findings to our results come from the research study by Virant-Klun et al. on small ovarian stem cells found on the ovarian surface epithelium of postmenopausal women with no naturally present oocytes and follicles. These small ovarian stem cells expressed the pluripotent markers SSEA-4, Oct-4, Nanog and Sox-2, and, in addition, they were detected to be also positive for c-kit, a receptor implicated in hematopoiesis and gametogenesis, suggesting that although pluripotent, these cells were also committed to specific lineages [191]. Finally, our results agree with a recent study that identified a novel population of stem cells (termed MUSE cells) isolated from peripheral blood that simultaneously express SSEA-3 and CD45 [192]. Although these MUSE cells were 10 µm, this leads further to the notion that indeed there is a primitive subpopulation of small stem cells that bears both pluripotent, mesenchymal and hematopoietic stem cell markers. On the other hand, all these findings on the different stem cell populations with different expressing markers, such as hematopoietic or mesenchymal ones, might suggest that there are circulating stem cell subpopulations at different stages of differentiation depending on the organism’s needs. For instance, Parte et al. found that there are two distinctive stem cell populations in the ovarian surface epithelium that differ in size, embryonic marker site expression and differentiation potential [193].

Apart from the various expressed markers, SBSCs appeared to differ from hematopoietic stem cells (HSCs) in terms of other characteristics as well. One of these characteristics was their population size found in peripheral blood; while most HSCs are increased in the bone marrow and limited in the peripheral blood [194,195,196], the SBSCs population seemed to be high in number in the peripheral blood. Another characteristic in regards to their size, SBSCs seem to be smaller than 5 μm, while the size of HSCs usually varies from 6 to 10 μm [197,198,199]. Finally, regarding their nucleus-to-cytoplasm ratio, SBSCs appear to have very little cytoplasm with nuclear Oct-4 staining, as shown in our study, while HSCs have larger cytoplasm and cytoplasmic Oct-4 staining [200].

Finally, although our results indicate that the isolated SBSCs express various pluripotent markers, suggesting that they are a mixed stem cell population, further characterization is required. Flow cytometry analysis and cell sorting would be an interesting approach, as these methods would not only further characterize this cell population, but could also compare the SBSCs’ expression profile with that of other stem and non-stem cells and provide information about the subpopulations’ actual cell number size. Apart from their protein content, another significant approach would be to analyze and compare their transcriptomic profile with other differentiated and non-differentiated cell populations, and this could be achieved using next-generation sequencing methods.

## 5. Conclusions

In this study, we presented an easy process to isolate small peripheral blood pluripotent stem cells with a strong pluripotent stem cell identity, while also sharing certain mesenchymal and hematopoietic characteristics. Although this population was morphologically similar to the VSELs, they were characterized as SBSCs due to the expression of different markers, as well as having different sizes and isolation methods. Proteomic analyses provided not only a composition analysis of SBSCs (stem cell markers CD9, MAPK1, STAT3, cell surface markers and signaling molecules) but also showed how specific factors were enriched in comparison to control blood cells. Currently, almost every VSEL isolation method requires the use of density gradients and erythrocyte lysis reagents that ultimately result in few isolated stem cells, while our approach uses only two centrifugation steps and no extra reagents and results in a rich stem cell population. VSELs may be a subgroup of SBSCs as SBSCs were found in higher quantity and mostly CD45 positive; however, further studies on the role and nature of SBSCs are crucial. The findings of the present study do not explain the functional role of SBSCs; whether they are a stand-alone cell population or a population of early tissue progenitor cells in blood that move from blood to tissue, and vice versa, is the subject of future work. Additional work is also necessary to determine their self-renewal and differentiating capacity into ectodermal, endodermal and mesodermal tissues and to examine their possible role in regenerative medicine applications. Furthermore, in terms of future diagnostic and therapeutic use, which is the main focus in our laboratory, it is plausible to suggest that these cells have the potential to be a natural source of adult pluripotent stem cells that could fulfill the need for an unlimited supply of autologous pluripotent stem cells that could be manufactured as end-products for personalized diagnostics and immune-rejection free autologous therapeutics.

## Figures and Tables

**Table 1:** List of antibodies used for the characterization of SBSCs.

Pluripotency	Antigen
Embryonic	Nanog ^1^, Oct4 ^1^, CXCR4 ^1^, SOX2 ^1^, KLF4 ^1^, cMyc ^1^, SSEA-3 ^1^ and SSEA-4 ^2^
Mesenchymal	CD29 ^3^, PTH1R ^1^, CD105 ^1^ and CD106 ^3^
Hematopoietic	CD45 ^3^, CD34 ^1^, CD90 ^1^ and CD133 ^1^

^1^ Novus Biologicals, Littleton, CO, USA, ^2^ Merck Millipore, Burlington, MA, USA, ^3^ R&D Systems, Minneapolis, MN, USA.

**Table 2:** Identified proteins that are associated with stem cell differentiation processes.

Protein	Functional Role	Role in Stem Cell Differentiation
LIMS1	Adaptor protein that binds to integrin-linked kinase and has a vital role in the processes of cell adhesion [125,126,127]	Promotes chondrogenesis, differentiation of placental mesenchymal cells to smooth muscle cells and plays a crucial part in skeletal myogenic differentiation [128,129,130]
HSP90AB1	Molecular chaperone involved in protein degradation [131,132,133]	Plays a role in the endoderm differentiation towards hepatic cells [134] and in mesodermal differentiation [135]
EIF5A	Associated with protein translation and cell proliferation and viability [136,137,138]	Supports embryonic stem cell differentiation and promotes skeletal muscle stem cell differentiation [139,140]
LYN	Belongs to the Src-family of tyrosine kinases and it is a nonreceptor cytoplasmic protein. It has a pivotal role in various cellular processes such as migration, cell growth, apoptosis, adhesion, differentiation, metabolism and immune response [141,142,143]	It has significant role in the differentiation process of embryonic stem cells [144,145,146]
S100A4	Belongs to the S100 family of calcium-binding proteins and is involved in various processes, such as angiogenesis, cell growth, motility, differentiation, apoptosis and invasion [147,148,149,150,151]	Promotes the differentiation of endoderm towards cardiomyocytes [152] and has a central role in the epithelial-to-mesenchymal transition [153]
PA2G4	It is a protein regulator of the ErbB3 signaling pathway, and it plays a role in cell growth, apoptosis and differentiation [154,155,156]	It plays a significant role in the differentiation of muscle stem cells and pluripotency of stem cells [154,157]
FYN	Belongs to the Src-family of tyrosine kinases and it is involved in various processes, such as cell growth, survival, adhesion, motility and tumor metastasis [142,143,158]	It is implicated in the differentiation process of mesenchymal stem cells [159] and has a significant role in the self-renewal of murine embryonic stem cells [145]
CAMK1	It is a protein kinase, primarily expressed in nerve cells, with various functions [160,161,162]	Unknown
PTBP1	Belongs to the heterogeneous nuclear ribonucleoprotein family and has a pivotal role in neuronal development [163,164,165]	It plays a significant role in the self-renewal of hematopoietic stem cells [166] and in the neuronal development [167]

## Data Availability

The data used to support the findings of this study are available from the corresponding author upon request.

## Supplementary Materials

The following supporting information can be downloaded at: https://www.mdpi.com/article/10.3390/biomedicines11030787/s1, Figure S1: Staining with isotype controls; Figure S2: SBSCs stained against the various targets of interest; Figure S3: H1 embryonic stem cell line stained for embryonic stem cell markers; Figure S4: SBSCs and WBC stained for CD45; Table S1: SBSC proteome; Table S2: SBSC functional enrichment analysis; Table S3: SBSC and control plasma cell proteome comparison.
