# Supplementary figures and images for: A Simplified and Effective Approach for the Isolation of Small Pluripotent Stem Cells Derived from Human Peripheral Blood
Source: Biomedicines. 2023 Mar 5;11(3):787. doi: 10.3390/biomedicines11030787 (PMC10045871; doi:10.3390/biomedicines11030787)

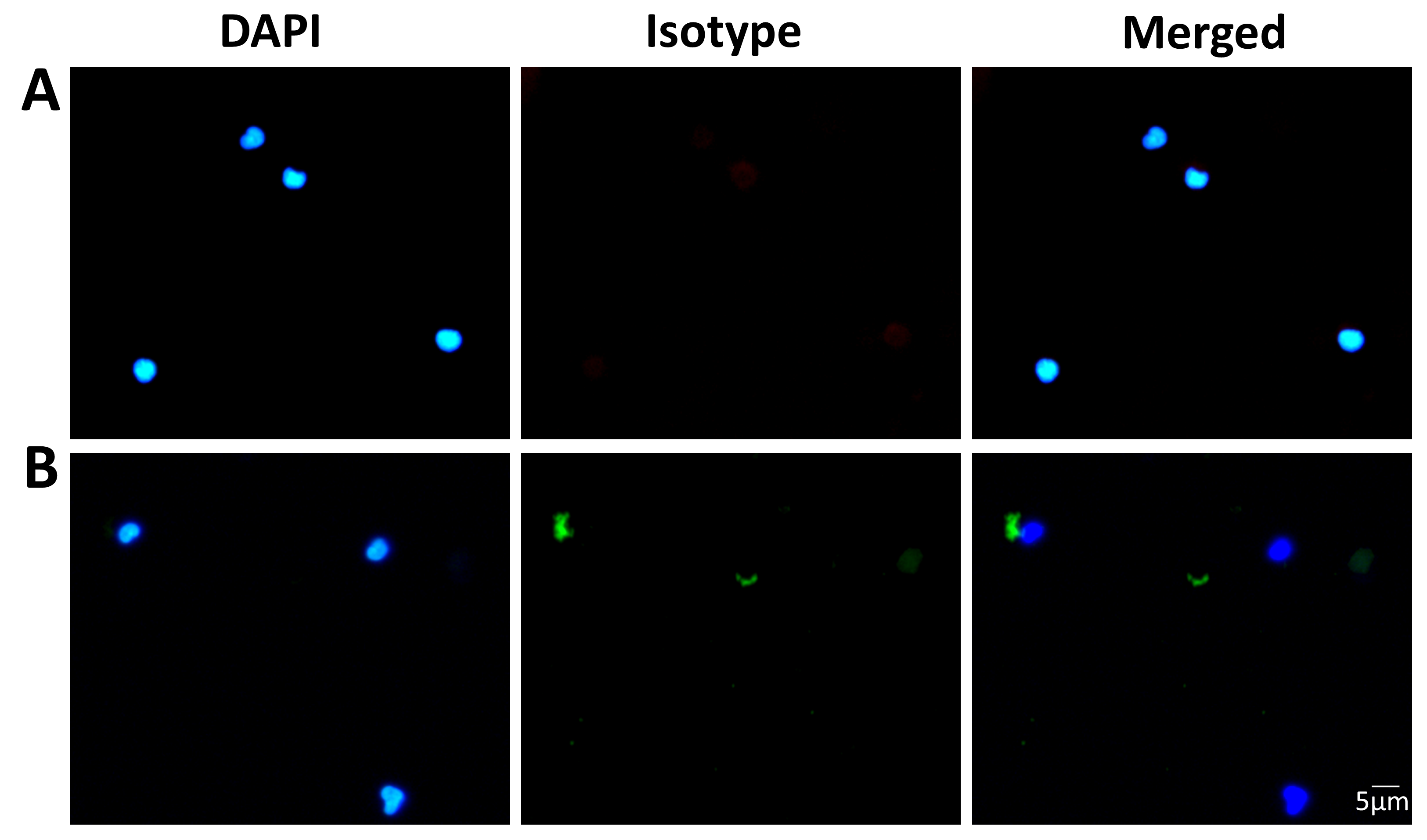

Supplement: Supplementary file 1 [file biomedicines-11-00787-s001.zip › Supplementary Figure S1.tif]

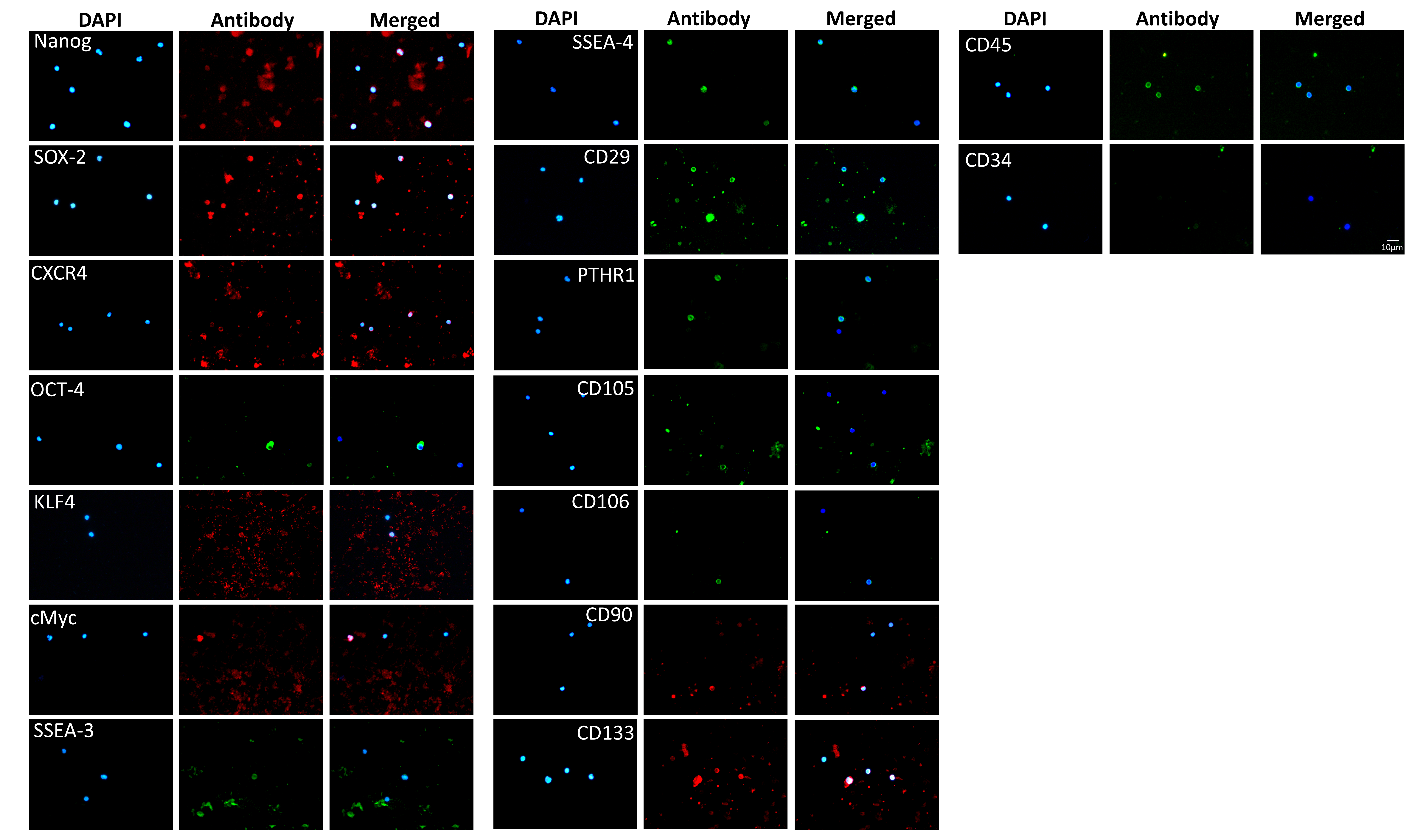

Supplement: Supplementary file 1 [file biomedicines-11-00787-s001.zip › Supplementary Figure S2.tif]

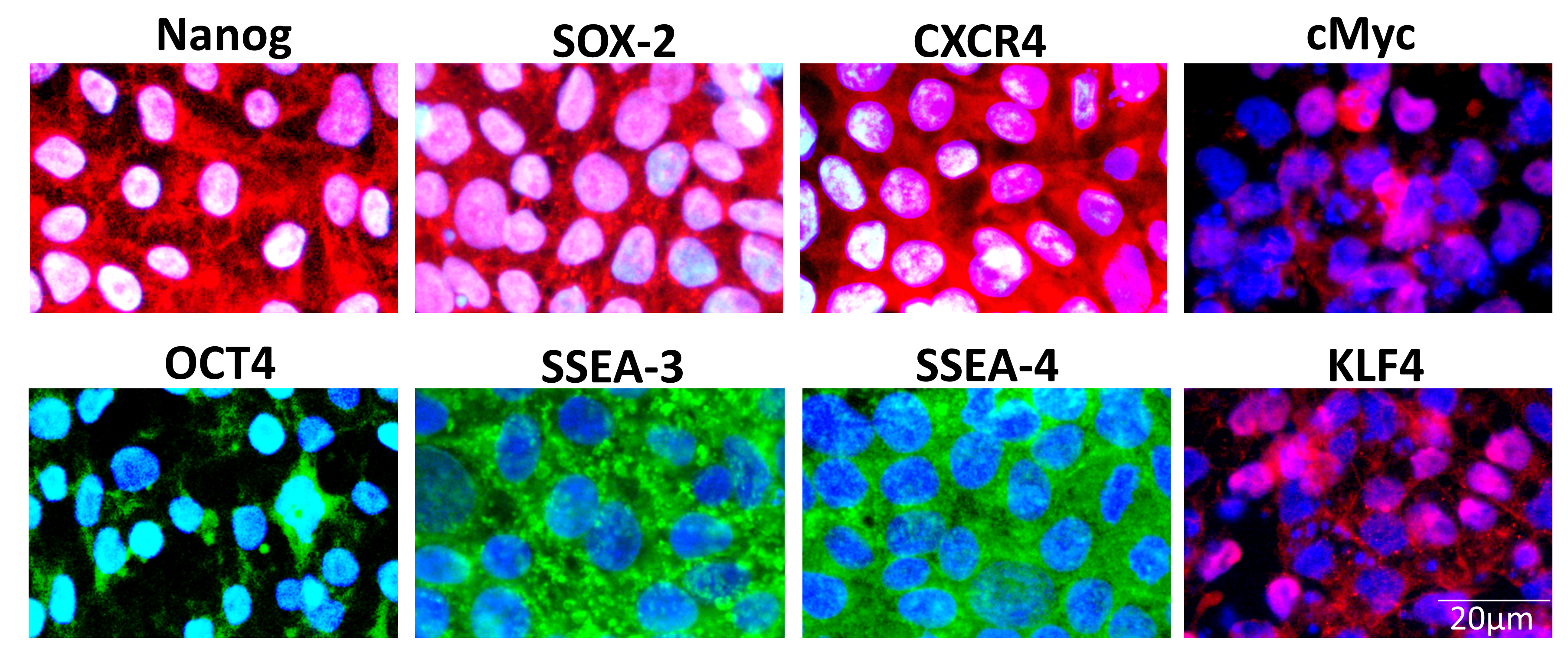

Supplement: Supplementary file 1 [file biomedicines-11-00787-s001.zip › Supplementary Figure S3.tif]

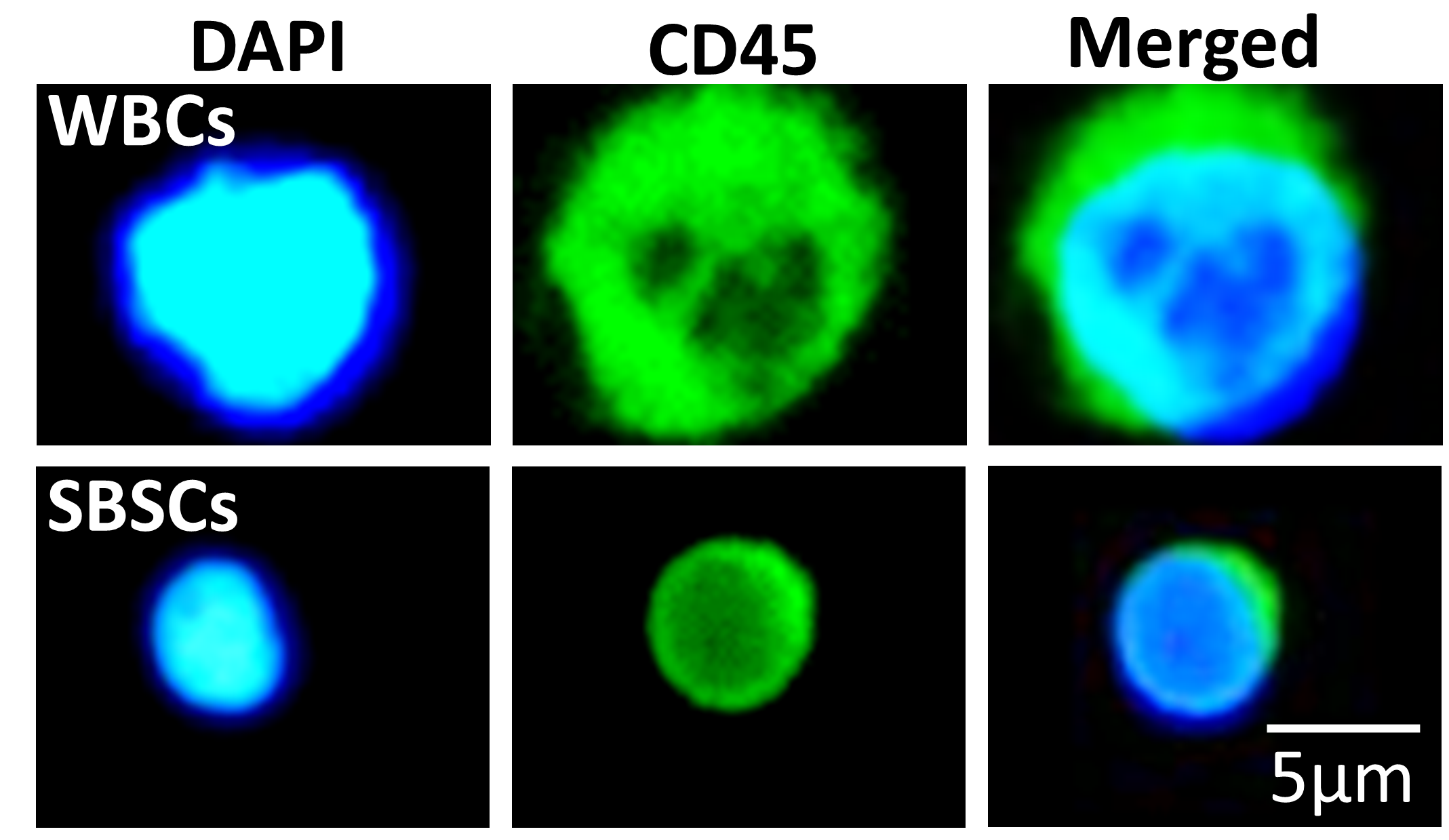

Supplement: Supplementary file 1 [file biomedicines-11-00787-s001.zip › Supplementary Figure S4.tif]
